# Adult depression screening in Saudi primary care: prevalence, instrument and cost

**DOI:** 10.1186/1471-244X-14-190

**Published:** 2014-07-03

**Authors:** Waleed Al-Qadhi, Saeed ur Rahman, Mazen S Ferwana, Imad Addin Abdulmajeed

**Affiliations:** 1 Board Eligible Resident, Family Medicine Department, King Abdulaziz Medical City-National Guard, Riyadh, Saudi Arabia; 2 Consultant Community Medicine, Family Medicine Department, King Abdulaziz Medical City-National Guard, Riyadh, Saudi Arabia; 3 Family Medicine Department, CoDirector- National & Gulf Center for Eveidance Based Health Practice, King Abdulaziz Medical City-National Guard, Riyadh, Saudi Arabia; 4 Staff physician, Family Medicine Department, King Abdulaziz Medical City-National Guard, Riyadh, Saudi Arabia

**Keywords:** Screened depression, PHQ-9, Cost-analysis, Primary health care, Saudi Arabia

## Abstract

**Background:**

By the year 2020 depression would be the second major cause of disability adjusted life years lost, as reported by the World Health Organization. Depression is a mental illness which causes persistent low mood, a sense of despair, and has multiple risk factors. Its prevalence in primary care varies between 15.3-22%, with global prevalence up to 13% and between 17-46% in Saudi Arabia. Despite several studies that have shown benefit of early diagnosis and cost-savings of up to 80%, physicians in primary care setting continue to miss out on 30-50% of depressed patients in their practices.

**Methods:**

A cross sectional study was conducted at three large primary care centers in Riyadh, Saudi Arabia aiming at estimating point prevalence of depression and screening cost among primary care adult patients, and comparing Patient Health Questionnaires PHQ-2 with PHQ-9. Adult individuals were screened using Arabic version of PHQ-2 and PHQ-9. PHQ-2 scores were correlated with PHQ-9 scores using linear regression. A limited cost-analysis and cost saving estimates of depression screening was done using the Human Capital approach.

**Results:**

Patients included in the survey analysis were 477, of whom 66.2% were females, 77.4% were married, and nearly 20% were illiterate. Patients exhibiting depressive symptoms on the basis of PHQ9 were 49.9%, of which 31% were mild, 13.4% moderate, 4.4% moderate-severe and 1.0% severe cases. Depression scores were significantly associated with female gender (p-value 0.049), and higher educational level (p-value 0.002). Regression analysis showed that PHQ-2 & PHQ-9 were strongly correlated R = 0.79, and R2 = 0.62. The cost-analysis showed savings of up to 500 SAR ($133) per adult patient screened once a year.

**Conclusion:**

The point prevalence of screened depression is high in primary care visitors in Saudi Arabia. Gender and higher level of education were found to be significantly associated with screened depression. Majority of cases were mild to moderate, PHQ-2 was equivocal to PHQ 9 in utility and that screening for depression in primary care setting is cost saving.

## Background

Depression is a mental illness that causes persistent low mood and a sense of despair in the suffering person [1]. It makes a person feel sad, frustrated, hopeless, have low self-esteem, and lose interest in things one usually enjoys [2]. Between 45-95% (overall 69%) of patients with depression present with somatic symptoms [3]. Depression has multiple underlying risk factors such as chronic medical illness, stress, chronic pain, family history, female gender, low income, job loss, substance abuse, low self-esteem, lack of social support, past history, being single, divorced or widowed, traumatic brain injury, and younger age [4].

The prevalence of depression in primary care setting varies according to the subtype, with major depression at 4.8-8.6%, dysthymia between 2.1-3.7%, and minor depression around 8.4-9.7%. Cumulative prevalence for all types of depression is between 15.3-22%, for patients seen in primary care [5,6]. According to 2001 Health Report of WHO, nearly 15% of patients with major depression have lifetime risk of committing suicide [7], although recent estimates are as low as 4%. In the United States, depression prevalence has been reported around 9% in general population [4,8,9] and varying between 5-13% among adult patients visiting primary care [10]. In Europe, the overall prevalence reported is 8.5%, of which women average around 10% and men at 6.6% [11]. Globally prevalence of depression has been reported as increasing, in the last decade [12]. In developing countries, 10-44% suffer from depression and anxiety disorders, and less than 35% of the depressed receive medical care [13]. Pakistan has an overall prevalence of 34% [14,15]. In Qatar, the prevalence is 27.8% [16].

In Saudi Arabia, prevalence has been estimated in several studies, with rates varying in different populations, age groups, times, and geographic locations. Psychiatric morbidity in primary care was estimated in 1995 around 30-46% of the visiting patients [17]. In 2002, depression and anxiety disorders were noted around 18% among adults in central Saudi Arabia [18]. Al Ibrahim et al., in 2010 showed an overall prevalence of 41% in a systematic review on depression [19]. El Rufaie et al., noted a 17% prevalence of depression among residents of Dammam [20]. Al Qahtani et al., in Asir reported a 27% prevalence of depression in the year 2008 [21]. Abdul Wahid et al. in 2011, reported an overall prevalence of depression nearing 12%, with 6% as severe cases, in the south-eastern region [22]. In Riyadh Becker et al., found depression prevalence to be 20% in primary care settings [23,24].

WHO reported depression as the leading cause of disability as measured by years lost due to disability (YLD) and the 4th leading contributor to the global burden of disease, as calculated by (DALYs) Disability Adjusted Life Years; depression is already the second major cause of DALYs lost in the age category 15–44 years for both sexes combined [5,7,15]. At present nearly half of lost productivity in the United States is attributable to depression, an estimated $17 billion annual loss [9,25]. Saudi Arabia has a high prevalence of depression, and as population grows, along with rising risk factors of depression such as chronic disease, stress of modernization, sedentary life style and social isolation, coupled with pre-existing stigmas of having a mental health disorder, paucity of psychiatrist and resources supporting mental health, the direct and indirect costs of depression are expected to rise [26]. In Saudi Arabian health care system in general and primary care settings in particular, data regarding cost of treatment of depression are rare to find. No Saudi studies regarding the cost of treatment, lost productivity and/or monetary benefit of screening for depression were found upon literature review.

Not just in Saudi Arabia, studies in other countries show that primary care physicians fail to recognize depressive symptoms in 30-50% of patients with depression [27]. In the absence of specific protocol or screening tools, the physicians are less likely to explore somatic symptoms as having possible links to depression [28]. On the other hand, many patients think that doctors are for treating physical symptoms only [29]. Almost two thirds of patients with depression receive care in primary settings [30]. In a recent study, Van den Berg et al. in 2011, calculated that screening for depression is cost effective around 80%, even for sub threshold depression, to prevent major depression [31]. On the contrary, a Cochrane review found that routine depression screening had minimal effect on the management or outcomes of depression after six or 12 months of follow-up [32].

United States Preventive Services Task Force (USPSTF) has recommended screening elderly, adults and adolescents 12–18 years of age for depression [4,33,34]. Ultra-short screening instrument, Patient Health Questionnaire (PHQ-2) asking two simple questions about mood and anhedonia, is as effective as longer screening instruments, such as the Beck Depression Inventory (BDI) or Zung Depression Scale (ZDS) [32,35,36]. PHQ-9 is one of the most common instruments used for depression screening, and it is increasingly being used for confirmation of a positive PHQ-2 result. The PHQ-9 is valid, takes two to five minutes to complete [4,37,38].

On the financial and cost aspect, Barrett et al., in 2005 found that there is no evidence that screening in primary care populations is a cost effective strategy [39]. Valenstein et al. and Nease et al., found that one time screening with PHQ-9 is cost effective rather than the annual and periodic screening [38,40]. Whooley et al. in 2009, found screening to be cost effective in primary care setting, only if followed by a collaborative care program [41].

The primary objective of this study was to estimate point prevalence of screened depression in primary health care settings in Riyadh, Saudi Arabia on the basis of screening instruments. Secondary objectives include exploring association of screened depression with some risk factors, comparing screening questionnaires PHQ-2 and PHQ-9, conducting cost analysis for depression and estimating possible cost-savings of screening for depression.

## Methods

This cross-sectional study was conducted at King Abdul Aziz Medical City-National Guard (KAMC-NG) in Riyadh, Saudi Arabia, in three large primary care centers, serving a population having nearly 60 thousand adults. Patients between 18–65 years, who attended primary care centers for their regular visits and agreed to participate, were included in this study. Patients who had preexisting depression or were on anti-depressants and/or refused to participate in this study, were excluded. Based on prevalence of depression at 20% from other studies [17,23,24] with ± 5% accuracy, confidence interval of 95%, power of .8, a sample size of 482 was estimated using Piface software, 2004 version.

This study was approved by King Abdullah International Medical Research Center, Riyadh. Data were collected using PHQ-2 and PHQ-9 Arabic version validated questionnaires for depression screening [42]. Other relevant demographic and personal data were also collected including age, gender, level of education, work status, monthly income, past medical history, social habits and place of residence. The survey forms were distributed and collected between 1^st^ of June, 2012 till 31^th^ of August, 2012. Each day, the nursing staff distributed questionnaires to 2–5 patients in each center based on a random number for the day, matching with the last digit of medical record number. Verbal and written consent were obtained from the respondents, clarifying the main purpose of the study, the importance of the respondent views, thanking in advance and assuring strict confidentiality of the information. Patients without education were assisted by an Arabic speaking nurse, who read out the questionnaire to the patient, and documented responses. During the process all data were kept secure. Completed forms were forwarded to the data entry clerk, who entered the data in IBM-SPSS version 20.

The PHQ-2 and PHQ-9 (Table 1) were analyzed in terms of calculating the severity scores for each question, for presence of depression symptoms over the last 2 weeks. The score of severity of depression varied between 0 (not present at all), 1 (present in several days), 2 (present more than half the days) and 3 (present nearly every day). The severity score of PHQ-2 was calculated and ranged between 0–6 points. Also, the severity score of PHQ-9 ranged between 0–27 points. The scores for PHQ-9 were used to determine the presence of depression and its severity depend on the following score ranges: 1–4 minimal depression, 5–9 mild, 10–14 moderate, 15–19 moderate to severe, and 20–27 severe [43]. For statistical analysis in our study, a person with minimal score (1–4) on PHQ-9, was not considered has ‘depressed’ , and those with score ≥ 10 (moderate - severe) were categorized needing medical treatment for cost-analysis. For PHQ-2, presence or absence of depression was based on a score of 3 and above out of 6 on the screening instrument [44]. Criteria for diagnosis of Major Depressive Disorder based on DSM IV, was not used as gold standard or validation of PHQ-9 cutoff in this study, as the purpose of the study was to assess the value of screening instrument for depression in primary care setting, and capturing as many adult patients with depressive symptoms as reasonably possible.

**Table 1:** Patient health questionnaire PHQ 2* & 9: screening instrument for depression

**For last 2 weeks how often have you been bothered by any of the following problems?**	**Not at all**	**Several days**	**More than half days**	**Nearly everyday**
Little interest or pleasure in doing things*	0	1	2	3
Feeling down, depressed, or hopeless*	0	1	2	3
Trouble falling or staying asleep, or sleeping too much	0	1	2	3
Feeling tired or having little energy	0	1	2	3
Poor appetite or overeating	0	1	2	3
Feeling bad about yourself — or that you are a failure or have let yourself or your family down	0	1	2	3
Trouble concentrating on things, such as reading the newspaper or watching television	0	1	2	3
Moving or speaking so slowly that other people could have noticed? Or the opposite — being so fidgety or restless that you have been moving around a lot more than usual	0	1	2	3
Thoughts that you would be better off dead or of hurting yourself in some way	0	1	2	3
If you checked off any problems, how difficult have these problems made it for you to do your work, take care of things at home, or get along with other people?	Not at all difficult	Somewhat difficult	Very difficult	Extremely difficult

The data was analyzed for all questions estimating frequencies, percentages, means and standard deviations, where applicable. The PHQ-9 scores were used along with various demographic variables, for comparisons, using statistical tests including Chi-square, Mann–Whitney Test, and Kurskal-Wallis Test. Relationships between PHQ 2, PHQ9 and “impact on daily life” question were explored, using linear regression.

### Cost analysis procedure

A simple cost-analysis was done by estimating direct and indirect costs of depression, followed by an estimate of possible cost-savings that may emerge as a beneficial outcome of improved screening and management of depression.

The following essential data were used from the current study to insert in calculations for cost-analysis: Percentage of males and females in the study, age range of patients, who were primarily adults in their productive life 18–65 years, marital status, and percentage of housewives. Average monthly income of SAR 93,472/year, rounded to SAR 8000/month ($2133) in Saudi Arabia was also used, which was quite close to monthly income average of study population [45].

In addition, percentages related to depressive symptoms from the current study were included for cost-analysis. For calculations, patients with mild symptoms were not considered for treatment or cost-analysis, those with moderate and severe symptoms of depression were grouped together and rounded to 20% and considered eligible for medication prescribed either by the family medicine physician or a psychiatrist. Psychotherapy was not added to the costs, due to infrequent availability of the service in the region, and variability in practice which might have made cost estimates fluctuate unreasonably. Literature review shows that nearly 45% of patients with major depression receive treatment, while most patients with major depression are managed by primary care physicians, only 5% get referred to a psychiatrist and 1% get admitted for treatment of depression [40]. These percentages were utilized in cost-analysis.

Lifetime risk of suicide varies between 4-15% of patients with major depression, and up to 3.6% of the depressed patients with or without receiving treatment in primary care for major depression are likely to commit suicide every year [46]. For conservative estimates in calculations, the suicide incidence rate was reduced to 1% of the patients with moderate-severe symptoms of depression (0.2% of total), screened in this study. In order to estimate indirect costs due to depression, the loss in productivity was estimated based on available data, that on the average, a depressed individual is absent from work no less than 18 days per year (rounded up to 20 workdays which is equivalent to one month of work), a care-giver for the depressed patient also takes 10 workdays off, and in addition, the depressed individual may show up for work, yet not work, an estimated loss of 11 days/year attributed to Presenteeism [47,48].

Human capital approach was used to estimate indirect costs. By this method a human being is valued by productivity. In estimating costs, only per year costs were calculated and using 1000 patients as a reference, all percentages were used to estimate number of patients affected within that pool. Valenstein et al. have used a direct cost-offset of 20% and a similar indirect cost-differential between patients treated for depression versus the untreated [40]. This difference was used to conduct sensitivity analysis, to come up with conservative estimates and other possible scenarios. Patient visits rate to physicians (general practitioners and psychiatrists) for depression, were derived from the work of Chisholm et al. [49]. Unit costs of physician fees and per diem cost of hospital stay were averaged based on current market charges in the private sector, and validated by local experts in the field of psychiatry and health administration.

Blood money is a compensation given to the family of the deceased, in case of accidental or homicidal death. Diyya (Blood Money) estimate has been derived by averaging homicidal and accidental death cost of 2011 revised rates [50]. This is based on the assumption that suicidal death although intentional yet can sometimes be accidental. For the sake of conservative estimate, Diyya was taken into account instead of the ‘life-time’ monetary loss of an earning person who commits suicide that can be extended to the whole span of productive life, with annual earnings discounted at 3-5%. With an average monthly salary of SAR 8000 ($2133), the loss would be in millions per person depending upon the age of suicidal death. The absentee rate was applied to a third of depressed patients assuming that only 1/3 of the population was employed. Similarly home-makers were estimated to be only half of the females in the study. Care-givers productivity losses were applied to both the employees. The home-makers’ earning were estimated based on the average monthly salaries of the persons doing various jobs in the house, i.e. housekeeping, cooking and babysitting. Other jobs were excluded from the analysis. A low total cumulative estimate of SAR 4000 ($1067) was used as monthly income, for all the job-types carried out at home.

## Results

A total of 550 questionnaires were distributed, 39 (7%) refused to participate, 23 (4.1%) were excluded from the study with pre-existing depression or affective disorders according to the medical records, and 11 (2%) incomplete questionnaires were rejected. The analysis included 477 patients; males were 161 (33.8%) and females were 316 (66.2%), 77.4% were married, with mean age of 38 years (±12 SD), and 50.9% were between 30–44 years. Study subjects living single were 108 (22.6%). Nearly 60% of patients had high school education, illiterate were 94 (19.7%), and those with degree above high school were 96 (20.1%). Those currently having an occupation were 259 (54.3%), housewives 45% and over 85% were living in a rented house. Reporting as smokers were 23 (4.8%), and coffee drinkers were 75.3%. The study found the point-prevalence of screened depression to be 49.9% among the adult visitors to primary healthcare, based on the predetermined cut-off limits on screening instruments. Of the screened depressed, mild were 31%, moderately depressed were 13.4%, moderate to severe 4.4% and severe were 1% (Table 2).

**Table 2:** Prevalence of depression among study subjects

**PHQ-9**						
**5 category scale**	**Minimal**	**Mild**	**Moderate**	**Moderately severe**	**Severe**	**Total**
No. of patients	239	148	64	21	5	477
% of patients	50.1	31.0	13.4	4.4	1.0	100
**2 category scale**	**Absent**	**Present**				
No. of patients	239	238				477
% of patients	50.1	49.9				100

**Figure 1:**
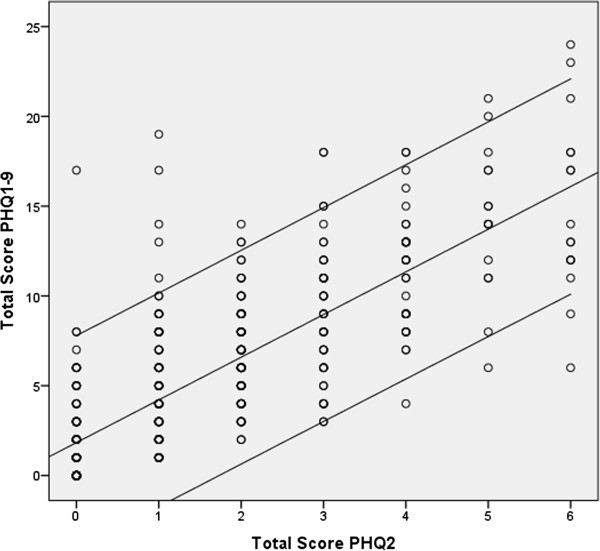
**Correlation between PHQ-2 & PHQ-9 scores.** R = 0.79, R^2^ = 0.62, F Statistic = 780.5, p-value <0.001. Regression equation: PHQ-9 = 1.83 + 2.37 x PHQ-2.

The number and percentage of patients responding to questions with various durations/severity of depression are given in Table 3. There was a significant relationship between prevalence of depression with female gender (Mann Whitney 22864, Z 1.97, *p-value 0.049*), and with higher level of education (Kruskal Wallis chi-square 14.9, *p-value 0.002*). No other significant relationships were found with other variables such as marital status, age, monthly income, working status, house space, house owner ship, chronic diseases, coffee intake and smoking among the study subjects.PHQ-2 and PHQ-9 were analyzed in term of calculating the severity scores for each question for the last 2 weeks. The severity score of PHQ-2 ranged between 0–6 points (mean of 1.57 ± 1.63SD) and that of PHQ-9 was found ranging between 0–27 points (mean of 5.57 ± 4.91SD) (See Figure 1). Cronbach’s alpha between PHQ-2 and PHQ-9 was 0.641 and on standardized items (2) was 0.882 (Figure 1).

**Table 3:** PHQ-2* & PHQ-9 Prevalence of screened depression in study subjects (477)

**No**	**Item**	**Number of patients in each category (%)**			
		**Not at all**	**Several days**	**More than half days**	**Nearly every day**
**1**	Loss of interest*	226 (47.4)	141 (29.6)	47 (9.9)	63 (13.2)
**2**	Feeling depressed*	257 (53.9)	146 (30.6)	42 (8.8)	32 (6.7)
**3**	**Trouble sleeping.**	242 (50.7)	128 (26.8)	62 (13.0)	45 (9.4)
**4**	**Feeling tired.**	150 (31.4)	199 (41.7)	68 (14.3)	60 (12.6)
**5**	**Poor appetite or eating.**	262 (54.9)	116 (24.3)	50 (10.5)	49 (10.3)
**6**	**Loss of self-esteem.**	376 (78.8)	77 (16.1)	12 (2.5)	12 (2.5)
**7**	**Low level of concentration.**	296 (62.1)	125 (26.2)	27 (5.7)	29 (6.1)
**8**	**Low voice or edgy.**	346 (72.5)	80 (16.8)	21 (4.4)	30 (6.3)
**9**	**Suicidal ideation.**	459 (96.2)	11(2.3)	4 (0.8)	3 (0.6)
**10**	**Feeling difficulty in general.**	265 (55.6)	199 (41.7)	10 (2.1)	3 (0.6)

PHQ 9 and PHQ 2 scores were also positively correlated to ‘impact on daily living’ with R = 0.49, R^2^ = 0.24, F Statistic = 150.3, p-value <0.001, and R = 0.42, R^2^ = 0.18, F Statistic = 102.2, p-value <0.001 respectively, with regression equations PHQ-9 = 3.6 + 4.2 (Q-10), and PHQ-2 = 1 + 1.2 (Q-10).

The cost analysis results are shown in Table 4.

**Table 4:** Cost of depression based on screening instrument PHQ-9 cut-off score ≥ 10

**Direct cost**	**Rating basis**	**Unit cost (SAR)**	Patients/1000*	**Total (SAR)**	**Comment**
PHC screening	Once/year	10	1,000	10,000	Use of PHQ-9 by nurse
GP visits	4 visits/year	100	90	36,000	45% needing treatment (90/200)
Specialist visits	8 visits/year	300	10	24,000	5% referred to psychiatrist (10/200)
Medication	Full year	200/month	90	216,000	45% on meds (90/200)
Hospitalization	11 days/year	1,500/day	2	33,000	1% hospitalized (2/200)
**Total**				**319,000**	$85,067*
					
**Indirect cost**	**Rating basis**	**Unit cost (SAR)**	**Patients/1000**	**Total (SAR)**	**Comment**
Suicide	Diyya 2011	350,000/life	2	700,000	Human life valued by ‘Blood Money’ rate
Absenteeism	20 days/year	8,000/month	30	240,000	20 work days equivalent to 1 month for 30/90 patients
Presenteeism	11 days/year	8,000/month	30	120,000	Average monthly salary in SA for 1/3 (30/90 patients)
Homemaker	20 days/year	4,000/month	30	120,000	Housemaid + babysitter + cook = Salary (30/90)
Care-giver	10 days/year	4,000/month	60	120,000	Conservative loss of care giver days
**Total**				**1,300,000**	$346,667
**Grand total**				**1,619,000**	$431,733
**Sensitivity analysis**					
Cost differential	20%	Untreated-Treated		323,800	$86,347
Saving/person				324	For 1,000 adults screened in PHC = $86
Decreasing indirect cost & direct cost by 50%				490,500	650,000- 159,000 = ½ million SAR
Saving/person				500	For 1,000 adults screened in PHC = $133

*20% patients with score **≥** 10, categorized as ‘depressed’ in the study (200/1000). **$1 = 3.75SAR.

## Discussion

Nearly half of the adults walking into primary care clinics had depressive symptoms, in our study. This finding is similar to that reported by Al Ibrahim et al., in their systematic review in 2010 [19]. Moderate or severe depression in our study totaled around 19%, which is a little higher than 12% reported by Abdul Wahid et al., in 2011 in primary care settings, but closer to reported by Becker et al., at 20% in 2002 [22,24] and by El Rufaie et al., 25 years ago at 17% [20]. Abdul Wahid et al., conducted their study in Sharurah, Southeastren region of Saudi Arabia, which is generally considered a rural area compared to the capital city, Riyadh. Several studies have shown that prevalence expected to be less in rural area comparing to urban area [11,15]. Another reason of this variation could be due to use of different scales for of screening depression in the studies, e.g. using Hospital Anxiety and Depression Scale (HAD), versus the PHQ scale [20]. Compared to general population, it can be assumed that primary care visitors are more likely to have depression, because of their health status. Al-Shammari et al., in 1999 studied depression in elderly and reported 39% prevalence with 8.4% of severe cases [51]. Comparing our study results with a study done in United States, we found our rates to be higher across the board; mild cases (31% vs 9.9%), moderate (13.4% vs 3.7%), moderate to severe cases (4.4% vs 1.4%), and severe (1% vs 0.5%) [52].

A significant relationship between depression and female gender was found. Similar relation was reported in many studies either local [18,20,22,23] or international [4,11,52]. In our study we also found significant relationship of depressive symptoms with higher level of education. No local study has noted this relationship previously or one found no differences between being literate or illiterate [21]. Many international studies however differ in results and found the opposite that depression was more likely to be associated with lesser educational level [15,53-56]. This could be due to high unemployment rate in Saudi Arabia, being a stressor for the educated. No other significant relationships were found with other variables, although, other studies have found depression to be associated with being in a younger age group [4,16,18,21], marital status specifically being divorced and widow [4,16,57], low monthly income [4], working status being employed [22] or losing the work [4], living in small house space [22], house owner ship and/or having chronic diseases [18]. In addition, no association was found with social habits such as coffee intake or smoking among study subjects.

The poor screening and diagnostic practices of depressed patients by primary care physicians [24,25] encouraged us to search a simple, reliable, efficient and easy to interpret tool. We chose the PHQ over many other modalities such as Beck Depression Inventory BDI, Rahim-Anxiety-Depression Scale RADS, and General Health Questionnaire GHQ for two reasons; Arabic version was validated by a study done on our population [24], and it had high sensitivity and specificity [4,38]. To further satisfy our needs to simplify, we explored the use of PHQ-2 by correlating with PHQ-9. Seeking psychiatric help is still considered as a stigma in the regional society. Psychiatrists are few and far between in Saudi Arabia. Professionals such as psychologists and behavior therapists are even less. Data are lacking on depression and on other psychiatric ailments. Individuals and the society as a whole are stressed in trying to adapt to pace of modernization. In retrospect not more than half a century ago, lifestyle of the majority of the population was nomadic and there was hardly any wealth. Mental health services are still not recognized as a pressing need of the time in Saudi society. Even though most of the health care structure is dependent on the government, the delivery of organized psychiatric services is moving at a faster pace in the private sector.

There is dearth of data regarding the cost of depression screening in Saudi population. Our simplified analysis showed that the total direct cost of care for a thousand persons screened for depression in primary healthcare setting was one third million SAR/year ($85,067) (Table 4). The indirect costs due to lost productivity came around 1.3 million SAR per year ($346,667) for a thousand persons screened in primary health care. This is a conservative estimate. A total loss due to depression was around 1.62 million SAR per 1000 persons screened ($431,733).

Based on the assumptions of another study [40], applying the cost differential of 20% savings in treated versus untreated patients with depression, revealed net cost saving of over a third of a million SAR, ($86,347) i.e. For every person who is walking in the PHC clinics, over 300 SAR ($80) can be saved per person per year, if screened and subsequently treated if required. Similarly, if it is assumed that treatment costs would go up by say 50%, as more patients are likely to received medical treatment for depression as screening becomes common practice, it is also assumed simultaneously that it would reduce indirect losses in productivity by a certain percentage, say 50%, as treated patients are less likely to be absent from work and coupled with reduced risk of suicide, it is estimated that over half a million SAR ($130,800) can be saved per thousand persons screened in the primary care clinics. This saving is up to SAR 500 per person ($133) screened in the clinic.

### Limitations

Our study has several limitations such as volunteer bias of including only those agreeing to participate, conducting study in summer so seasonal depression could not be included, based on primary care settings with more female and sick visitors thereby lacking generalizability, and cost estimates had no long term component and were based on several assumptions. In addition we did not use gold standard diagnostic criteria such as DSM IV for major depressive disorder rather relied on cutoff of PHQ-9 scores of ≥ 5 for point prevalence estimates for screened depression and score of ≥ 10 for cost-analysis estimates of those who may require treatment. This might have resulted in over-estimates of point prevalence of screened vs actual depression in primary care setting, but considering that nearly 30-50% of actual depression is missed, and of the depressed only 45% get treatment, our cost estimates may not be unreasonable. Our cost estimates are limited by several assumptions due to lack of resources on data related to healthcare costs, in the country.

## Conclusion & recommendation

Nearly half of the patients visiting primary care have some depressive symptoms, which require further exploration into their psychiatric history. The female gender and higher education level were factors associated with depression. Majority of the cases were mild to moderate cases. As screening tools, PHQ-2 and PHQ-9 correlate well and can easily be adopted in primary care. Simple cost estimates using basic percentages, using references from other studies and making some safe assumptions, it can be demonstrated that of screening for depression in primary care, is cost-saving.

Our results need further validation by conducting population based studies, or within primary care clinics with a larger sample size and confirmation of screened patients with some gold standard for measuring depression. It is also recommended to increase the awareness of benefits of early diagnosis of patients to prevent major form of depression and cost saving of health system. Programs for screening depression should be implemented in primary care settings.

## Competing interests

The study funded by King Abdullah International Medical Research (**KAIMRC**) at King Saud bin Abdulaziz University for Health Sciences (**KSAU**-**HS**) in National Guard Health Affairs (**NGHA**). There is no stocks or shares in an organization that may financially gain or lose from publishing this study. Moreover, there are no patents relating to the content of the manuscript. However, this study is a part of an educational process to complete research during the residency program of Family Medicine/Saudi Board under Saudi Commission for Health Specialties.

## Authors’ contributions

WQ conceived of the study, and participated in its design, coordination, and performed the statistical analysis. Moreover, WQ has drafted and revised the manuscript. SR contributed in the conception and designs the study, analyzing and interpreting of data. Also, SR has been involved in drafting the manuscript. MF has been involved in its design, interpretation of data and revising the manuscript critically for the final approval to be published. Also, recently AI has been involved in review and revising data and manuscript. All authors read and approved the final manuscript.

## Pre-publication history

The pre-publication history for this paper can be accessed here:

http://www.biomedcentral.com/1471-244X/14/190/prepub
